# Severe Cutaneous Leishmaniasis in a Human Immunodeficiency Virus Patient Coinfected with *Leishmania braziliensis* and Its Endosymbiotic Virus

**DOI:** 10.4269/ajtmh.15-0803

**Published:** 2016-04-06

**Authors:** Laurent Parmentier, Alexia Cusini, Norbert Müller, Haroun Zangger, Mary-Anne Hartley, Chantal Desponds, Patrik Castiglioni, Patrick Dubach, Catherine Ronet, Stephen M. Beverley, Nicolas Fasel

**Affiliations:** Department of Dermatology, University Hospital of Bern, Bern, Switzerland; Department of Infectious Diseases, University Hospital of Bern, Bern, Switzerland; Institute of Parasitology, Vetsuisse Faculty Berne, University of Bern, Bern, Switzerland; Department of Biochemistry, University of Lausanne, Lausanne, Switzerland; Department of Ear, Neck and Throat, Head and Neck Surgery, University Hospital of Bern, Bern, Switzerland; Department of Molecular Microbiology, Washington University School of Medicine, St. Louis, Missouri

## Abstract

*Leishmania* parasites cause a broad range of disease, with cutaneous afflictions being, by far, the most prevalent. Variations in disease severity and symptomatic spectrum are mostly associated to parasite species. One risk factor for the severity and emergence of leishmaniasis is immunosuppression, usually arising by coinfection of the patient with human immunodeficiency virus (HIV). Interestingly, several species of *Leishmania* have been shown to bear an endogenous cytoplasmic dsRNA virus (LRV) of the *Totiviridae* family, and recently we correlated the presence of LRV1 within *Leishmania* parasites to an exacerbation murine leishmaniasis and with an elevated frequency of drug treatment failures in humans. This raises the possibility of further exacerbation of leishmaniasis in the presence of both viruses, and here we report a case of cutaneous leishmaniasis caused by *Leishmania braziliensis* bearing LRV1 with aggressive pathogenesis in an HIV patient. LRV1 was isolated and partially sequenced from skin and nasal lesions. Genetic identity of both sequences reinforced the assumption that nasal parasites originate from primary skin lesions. Surprisingly, combined antiretroviral therapy did not impact the devolution of *Leishmania* infection. The *Leishmania* infection was successfully treated through administration of liposomal amphotericin B.

## Introduction

*Leishmania* parasites infect over 2 million people annually and cause a broad range of disease, with cutaneous afflictions being, by far, the most prevalent.^1^ Variations in disease severity and symptomatic spectrum are mostly associated with parasite species. This is reflected by the geographical distribution of severely disseminated (DL) and mucosal leishmaniases (ML), which occur predominantly in Central and South America and Ethiopia. *Leishmania braziliensis*, *Leishmania panamensis*, *Leishmania guyanensis*, and *Leishmania aethiopica* are the main causative species of these aggressive forms of leishmaniasis, which progress from a primary cutaneous lesion in 5–10% of infected patients.^2^,^3^

*Leishmania braziliensis*, *L. guyanensis*, and *L. aethiopica* species are known to be variously infected by *Leishmania* RNA virus (LRV), a cytoplasmic double-stranded RNA (dsRNA) virus of the *Totiviridae* family.^2^,^4^,^5^ LRV presence is predominant in the Amazonian basin.^6^ Previously, we showed that this virus (LRV1) within the *L. guyanensis* parasites exacerbates disease outcome in a murine model by inducing a hyperinflammatory response with increased interferon β,^7^ and inflammatory markers commonly found in the lesions of ML patients.^8^,^9^ Recently, we and collaborators showed that the presence of LRV1 in *L. guyanensis* and *L. braziliensis* is a risk factor for relapse in drug-treated patients.^10^,^11^

Leishmaniasis is a rising opportunistic complication in human immunodeficiency virus (HIV)–positive individuals.^12^ Although it is well known that the parasite can take advantage of the concomitant acquired immunodeficiency syndrome (AIDS)–related immunosuppression, potentially this could be further exacerbated by the presence of LRV1 within the infecting parasite. In this case report, we recount the clinical presentation, diagnosis, and successful treatment of this severe cutaneous leishmaniasis in an HIV-positive patient caused by *L. braziliensis* and its cytoplasmic virus.

## Case Report

**Figure 1.**
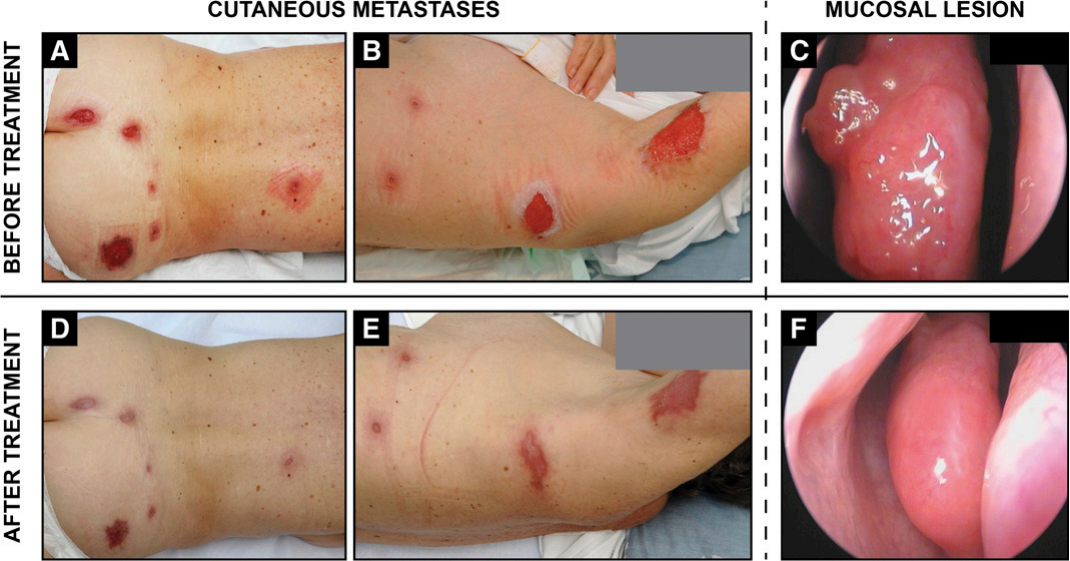
Cutaneous and mucosal metastatic leishmaniasis caused by *Leishmania* RNA virus–laden *Leishmania braziliensis* in a human immunodeficiency virus–coinfected patient. Pre-therapeutic examination (**A**–**C**) finds large well-demarcated skin ulcerations of the axillary regions and posterior trunk (**A** and **B**) while a nasal endoscopy (**C**) reveals granulomatous inflammation of the nasal mucosa on the head of the middle turbinate. One month after AmBisome treatment, skin lesions healed completely with mild discoloration and scarring (**D** and **E**). Nasal lesions resolved 3 months later after an additional round of AmBisome therapy (**F**).

The patient, a 60-year-old female, presented at the clinic with several skin ulcerations on the limbs and trunk. The patient had been living in Paraguay for the three previous years and her lesions first developed 9 months earlier during a 1-month stay in Bolivia, where she was treated empirically for bacterial ecthyma. A concomitant HIV test was found positive after which she decided to travel back to Switzerland for further medical management. The initial physical examination revealed several large, ovoid skin ulcerations, ranging from 5 to 15 cm in diameter, with raised erythematic borders and a clear exudate (Figure 1A and B

). The patient was apyretic and had no additional complaints.

Combined antiretroviral therapy (cART) with tenofovir, emtricitabine, and efavirenz was initiated based on a high viremia of 1,764 copies/mL (CD4-cell count of 803 cells/μL) and on the presence of a coinfecting *Leishmania* parasite. Treatment decreased viremia to 482 copies/mL after 2 months. The patient's skin lesions, however, continued to slowly progress, at which point she was referred to the dermatology department for further evaluation.

In addition to the skin lesions, the patient complained of a recently acquired unilateral rhinitis and nasal congestion. A nasal endoscopy revealed unilateral focal granulomatous hyperplasia of the head of the middle turbinate (Figure 1C). Magnetic resonance imaging showed no cartilaginous destruction or paranasal involvement. ML was suspected. Touch preparations from skin exudates disclosed no *Leishmania* amastigotes, and biopsies from lesion borders and nasal granulomatous tissue revealed chronic nonspecific inflammation, with no *Leishmania*, fungal, or mycobacterial organisms (by Giemsa, periodic acid–Schiff, and Ziehl–Neelsen stains, respectively).

On admission, a blood sample and two intralesional biopsies were isolated from the patient: one from a cutaneous lesion and the other from a metastatic lesion in the nasal mucosa. Two standard leishmanial diagnostic procedures (in vitro cultivation and polymerase chain reaction [PCR]) were undertaken on each of the three isolates. *Leishmania* parasites could be expanded from both cutaneous and nasal mucosa specimens, albeit absent in blood samples. PCR amplified a standard leishmanial diagnostic marker (internal transcribed spacer 1 of *Leishmania* small subunit ribosomal RNA).^13^ Direct sequencing of the amplified products was obtained and comparative sequence analysis of the diagnostic PCR products was done by applying the nucleotide BLAST^®^ algorithm (http://blast.ncbi.nlm.nih.gov/Blast.cgi) (GeneBank^™^ sequence database; accession no. JN936955). The alignment of the PCR products categorized both parasite isolates as *L. braziliensis*. Isolates were thereafter assigned World Health Organization format identifiers MHOM/BO/2011/2169 (*Lb*2169, skin lesion) and MHOM/BO/2011/2192 (*Lb*2192, nasal lesion).

Considering that our patient had a severely disseminated form of cutaneous leishmaniasis and an inflamed nasal mucosa, we determined that the *L. braziliensis* parasites from these lesions were infected by LRV1.^14^ We completed our study by obtaining pure LRV1 dsRNA after reverse transcription into complementary DNA (cDNA), performing PCR amplification using 10 primers (information available at: http://people.unil.ch/nicolasfasel/supptab1_lbhiv/) and sequencing 4,969 bp of this dsRNA (GenBank accession no. KC862308). Its structure paralleled the open reading frames of previously sequenced LRV1 isolated from *L. guyanensis* parasites (data not shown). An LRV1-specific PCR of parasite cDNA preparations from isolated viral dsRNA of both isolates was sequenced, confirming LRV1 presence in both isolates. The genetic identity of LRV1 between both isolates supports the assumption that mucosal parasites had migrated from primary skin lesions.

Although the standard first-line treatment of ML relies on pentavalent antimony (sodium stibogluconate or meglumine antimoniate),^15^,^16^ we opted for a second-line therapy with liposomal amphotericin B,^17^ whose efficacy and tolerance profiles are favorable in DL as well as in HIV-coinfected patients and for treatment of antimony-resistant strains.^18^ Our choice was motivated by the evolution and severity of the disease, where numerous, large diameter (up to 15 cm) skin lesions had evolved and persisted for over 10 months, together with the potentially disfiguring involvement of the nasal mucosa.

The patient received intravenous liposomal amphotericin B for five successive days at 3 mg/kg (150 mg per infusion) with two additional infusions at days 14 and 21 (cumulative dose: 21 mg/kg, i.e., 1,050 mg). The cutaneous lesions of the limbs and trunk began to heal 2 weeks after treatment initiation and were completely re-epithelialized after 1 month (Figure 1D and E). The nasal mucosa required additional liposomal amphotericin infusions (cumulative dose: 27 mg/kg, i.e., 1,350 mg) to achieve complete healing with no residual deformation of the nasal structure (Figure 1F). Six months posttreatment, the patient had no infectious relapse and displayed only mild discoloration of the healed wounds.

## Discussion

This study was the first account of a patient coinfected with HIV and a strain of *L. braziliensis* carrying the cytoplasmic dsRNA virus, LRV1. The patient presented with a particularly exacerbated form of cutaneous leishmaniasis, with individual metastatic skin lesions measuring up to 15 cm in diameter as well as a further parasitic infestation in the nasal mucosa. Although DL may present with ulcers and/or mucosal involvement, even in HIV-negative patients,^19^–^21^ this patient's symptomology was distinct from that of *L. braziliensis* DL^21^ and from HIV/*L. braziliensis* coinfections because of the number of large ulcerated disseminated lesions.^22^ Although exacerbations of leishmaniasis are common in HIV coinfection due to a collapse of the CD4 T-cell compartment, the patient described here had a low CD4-cell count at the start of cART. Further, cART had no effect on disease progression, contrary to visceral leishmaniasis/HIV coinfection, where restoration of the CD4-cell count usually results in a significant amelioration of disease. Quite oppositely, the patient condition did not improve clinically with progressing cutaneous lesions.

The presence of *Leishmania* along with two viruses (one internal, one external) could underlie this severe form of disease, and may potentially explain some of the unusual manifestations of American tegumentary leishmaniasis caused by *L. braziliensis* or *L. guyanensis* reported in HIV-positive patients,^23^,^24^ which are especially frequent in regions of South America where LRV1 is known to be most prevalent, such as French Guyana and the Brazilian Amazon.^25^,^26^ Indeed, HIV and LRV dsRNA may synergize to worsen leishmaniasis, where HIV coinfection not only increases the incidence of *Leishmania* infection, but also the patient's risk of developing metastatic or chronic complications of leishmaniasis.

The retrospective nature of this report and the lack of epidemiological data did not allow us to definitively ascertain the joint detrimental roles of LRV1 and HIV in human leishmanial disease progression. However, we can hypothesize that the clinical presentation reported here of LRV1-carrying *Leishmania* infection is in line with what is observed in relapses in *L. braziliensis*- and *L. guyanensis*-infected patients and in experimental animal models and could explain some of the unusual manifestations of American tegumentary leishmaniasis reported in HIV-positive patients,^23^,^24^,^27^ as well as the commonly observed treatment failures and relapses.^10^–^12^,^26^ Using PCR conditions and primers described in this study, a large-scale screen for LRV1 as a risk factor in leishmaniasis patients will be instrumental to unlocking the potential of exploiting this nested viral infection for use as a diagnostic and prognostic marker.
